# Approaching mercury distribution in burial environment using PLS-R modelling

**DOI:** 10.1038/s41598-021-00768-8

**Published:** 2021-10-27

**Authors:** Noemi Álvarez-Fernández, Antonio Martínez Cortizas, Zaira García-López, Olalla López-Costas

**Affiliations:** 1 CRETUS, EcoPast (GI-1553), Universidade de Santiago de Compostela, 15782 Santiago, Spain; 2 EcoPast (GI-1553), CRETUS, Archaeology Department of History, Universidade de Santiago de Compostela, 15782 Santiago, Spain; 3 Archaeological Research Laboratory, Wallenberglaboratoriet, Stockholm University, 10691 Stockholm, Sweden; 4 Laboratorio de Antropología Física, Facultad de Medicina, Universidad de Granada, 18012 Granada, Spain

**Keywords:** Element cycles, Geochemistry, Environmental impact, Natural hazards, Palaeoecology

## Abstract

Mercury environmental cycle and toxicology have been widely researched. Given the long history of mercury pollution, researching mercury trends in the past can help to understand its behaviour in the present. Archaeological skeletons have been found to be useful sources of information regarding mercury loads in the past. In our study we applied a soil multi-sampling approach in two burials dated to the 5th to 6th centuries AD. PLRS modelling was used to elucidate the factors controlling mercury distribution. The model explains 72% of mercury variance and suggests that mercury accumulation in the burial soils is the result of complex interactions. The decomposition of the bodies not only was the primary source of mercury to the soil but also responsible for the pedogenetic transformation of the sediments and the formation of soil components with the ability to retain mercury. The amount of soft tissues and bone mass also resulted in differences between burials, indicating that the skeletons were a primary/secondary source of mercury to the soil (i.e. temporary sink). Within burial variability seems to depend on the proximity of the soil to the thoracic area, where the main mercury target organs were located. We also conclude that, in coarse textured soils, as the ones studied in this investigation, the finer fraction (i.e. silt + clay) should be analysed, as it is the most reactive and the one with the higher potential to provide information on metal cycling and incipient soil processes. Finally, our study stresses the need to characterise the burial soil environment in order to fully understand the role of the interactions between soil and skeleton in mercury cycling in burial contexts.

## Introduction

Mercury is a global pollutant that is released to the environment from both anthropogenic and non-anthropogenic sources. Given its chemical properties, once it is available in ecosystems it is prone to bioaccumulation and biomagnification in food chains^1,2^. Mercury is considered one of the most potentially toxic metal elements^3^. There is no biological known function for it and it is harmful even at very low doses. This is the reason why the WHO considers mercury as an element of global public health concern^4^ and its toxicology and environmental cycling have been widely researched and monitored.

Mercury toxicology is complex, as it has different chemical species that do not necessary share absorption pathways neither behaviour inside the organism^5,6^. This variability leads to a wide range of target organs and clinical symptomatology, which can also be affected by dose, time, and route of exposure^7^. Mercury acute intoxication can damage permanently the nervous system, cause renal toxicity, myocardial infraction, immune malfunction, and irregular blood pressure, or in the worst of the cases it can lead to death^8^. However, acute intoxication with mercury is rare and the main health risk is related with chronic exposure. Chronic exposure is one of the major concerns among other reasons because of the damage that can cause in embryonic development through its impact in central nervous system formation^8^. Acute and chronic/environmental exposure have normally different target organs. When exposure is due to environmental levels, mercury mainly accumulates in kidneys and liver^9^, while in acute or occupational exposure high concentration can also be found in the central nervous system^10^.

Although most of the population is exposed to low-dose chronic mercury levels (i.e. environmental), mercury toxicology studies have been carried out mostly on high-level acute effects^11^. Severe mercury poisoning, also known as Minamata disease, has visual signs and symptoms that develop quickly. For years, the main interest has been focused in monitoring acute mercury poisoning, to eliminate direct sources of pollution. However, and once this problem has been more addressed, research has begun to consider chronic and environmental exposure in its focus. At this time, it has been realized that even at low doses, exposure has serious consequences for health, direct ones or by increasing the symptoms and severity of other ailments. Research dealing with the consequences of long-term and low-level exposure, primarily related with the environment, is still scarce. Low environmental levels may be also ultimately linked—in most cases—with anthropogenic sources, which means that the environmental impact of humans and their health consequences are closely related. This cause-effect interaction has been proposed as a promising tool in Environmental Health studies^12^. We have also indicated the interest of considering this interaction in the past^13^, to improve our knowledge about health consequences of mercury environmental pollution along history.

Mercury patterns and trends in the past have been studied through natural archives, among others lake and marine sediments, peat, and tree rings (see a review in^14^). They show that anthropogenic mercury pollution dates back at least to 3250 BCE (Copper Age) from South Iberian Peninsula^15^, while the first evidence of mercury atmospheric pollution dates back to c. 1400 BCE at Peruvian Andes^16^. There is also evidence of mercury mining in Almadén (SE Spain), the largest mercury mine known in Antiquity, from BCE 5300^17^. In our region of study (NW Spain), a reconstruction of the atmospheric mercury levels suggested that atmospheric mercury pollution started by 500 BCE^18^, right near the onset of Iron Age mercury mining in Spain. This work also reveals a more intense impact of the Roman Empire economy in atmospheric mercury pollution—due to extensive mining-metallurgy—and a later decreased after the Germanic invasions (post-Roman period, i.e. where our samples belong). Anthropogenic mercury atmospheric pollution showed a steady increase from the Islamic period (8th to 11th centuries CE) until the Modern era, when pollution increased exponentially. This research^18^ and later investigations using short peat cores spanning the last 150 years^19^, showed that the highest pollution levels occurred during the Industrial Period (i.e. last 300–350 years), reached a maximum around 1980s CE and then started to decreased. However, the analysed archives provide little information about the direct impact of mercury pollutant on biota, which is the most affected by mercury toxicity. Recently, we have proposed archaeological human remains as suitable archives^13,20^ to investigate the impact that changing mercury loads to the environment had in past-populations, especially on pre-industrial times. These works^13,20^ show the same trend previously found by^18^ in their paleo-reconstruction of Hg atmospheric levels, with Roman populations showing significantly higher mercury (and Pb) concentrations in skeletal remains than post-Roman populations. These studies confirm post-Romans times as a period of low environmental Hg levels. Nonetheless, preserved archaeological human remains are mainly skeletons that have been buried for long periods of time—much longer than the individual’s life. At this point, two aspects have to be considered: (1) bones are not the target organ for mercury, and (2) bones can be affected by diagenetic processes in the burial environment.

One of the major issues when studying trace elemental composition in archaeological human remains is diagenesis^21^. Where does the studied element come from? Was its incorporation *post-mortem* or *pre-mortem*? These issues must be also considered when researching mercury. Mercury can be absorbed during life through different ways depending on mercury chemical species and path of exposure^5,6^—skin contact, ingestion, inhalation, etc. Regarding *post-mortem* incorporation, it can be related to funerary rituals involving mercury-containing pigments^22^, from soil in burial areas with mercury ores^23^ or by transfer from target organs during body decomposition. To our knowledge, there is a handful of studies that deal with mercury in past populations^13,20,22–41^, and only a few assessed mercury diagenesis in burial context^13,22,23,31,36,41^ by analysing punctual soil samples associated with the bones and graves. ^42^approached mercury distribution vertically and horizontally in soil with respect to femur in three burials (n_v_ = 5–6, n_h_ = 5–6, per individual) in a medieval cemetery in Denmark. They found that there was no evidence of diagenetic incorporation of mercury in bone from the soil. However, to our knowledge, there are no systematic studies that analyse detailed transects throughout the body. Previous works also do not consider the variation in Hg content in relation to sources and soil components involved in Hg retention. Mercury in these studies is usually explained alone, ignoring the importance of the relationship between elements in geochemical studies. Since bone is not a mercury target, as it mainly accumulates in liver and kidney^5^, a question remains opens: how is mercury distributed in burials contexts and which are the factors controlling its distribution?

The current research aims to answer this question by modelling mercury distribution in the soil/sediments of two post-Roman-Early Medieval burials—one single (T1 with the skeleton L01) and one double (T5 with the skeletons L06 and L07)—from the archaeological site of A Lanzada in NW Spain. We used partial least square regression modelling (PLSR) for the first time in analysing mercury from archaeological soils. For this purpose, soil/sediment samples were collected inside and outside two burials. Fine earth and silt + clay fractions have been analysed from two transects in each skeleton (W–E and N–S) intended to cover the more likely gradients of mercury concentrations regarding distance to target organs. We have done a multi-sampling research in a small area (46 samples from 5 transect along 3 skeletons from two burials) to better address variability. The objectives of this modelling effort are: i) to elucidate how mercury soil concentrations vary inside and outside the burials; ii) how does burial context (single/multiple burial) affects total mercury content in soil; and iii) which other factors are involved in mercury release and retention in the soil/sediment.

## Results

The fine earth fraction samples showed very low Hg concentrations (median = 2.6 ng g^−1^, IQR = 1.7 ng g^−1^, relative error average = 47%). Mercury concentrations in the silt + clay fraction varied from 14 to 24 ng g^−1^ for L01, 12 to 39 ng g^−1^ for L06 and 6 to 33 ng g^−1^ for L07 (Table 1). Thus, concentrations in the finer soil fractions (i.e. silt + clay) are up to tenfold the average of the fine earth mercury concentrations. Silt + clay mercury concentrations in samples outside the burials ranged between 6 and 23 ng g^−1^ and those inside the burials between 12 and 39 ng g^−1^.

**Table 1:** Summary of Hg concentration in silt + clay fraction per individual (ng g^−1^).

	Minimum	Median	Maximum	IQR
L01	14.33	19.72	23.88	5.45
L06	11.79	18.42	39.27	7.23
L07	6.41	20.62	33.49	9.07

**Figure 1:**
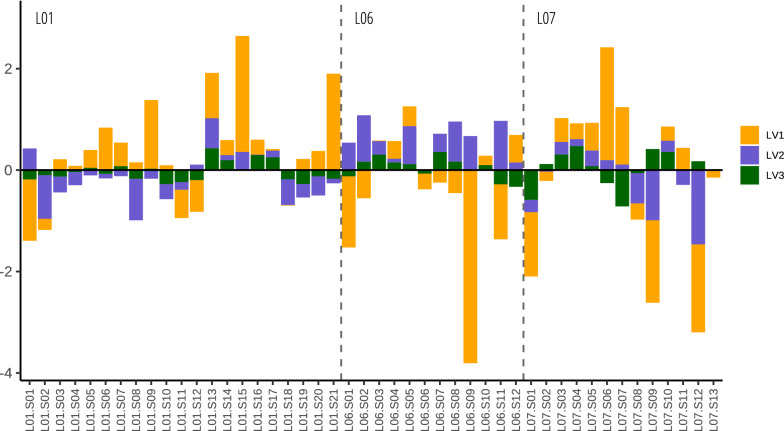
Latent variable predicted relative weight for each sample.

**Figure 2:**
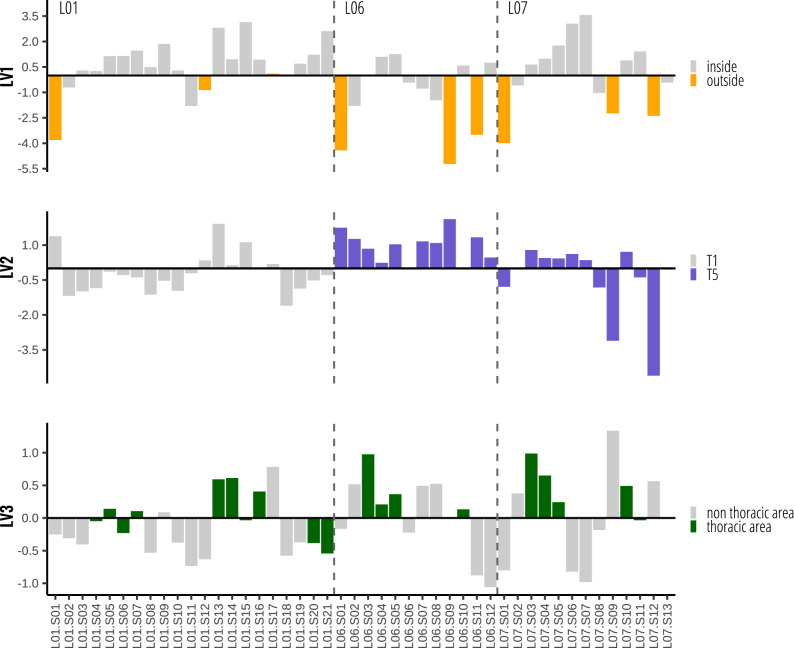
PLSR X-scores for each of the 3 LV.

**Figure 3:**
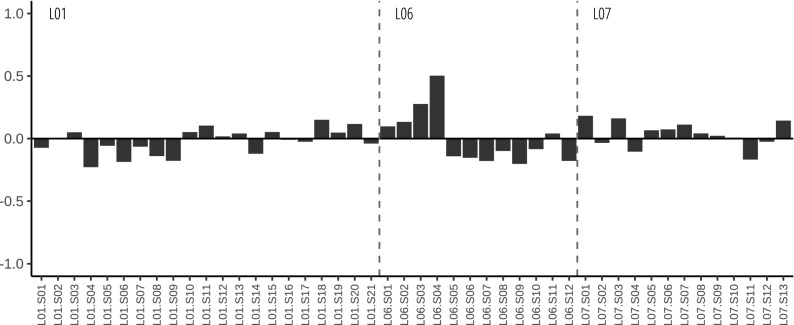
PLSR model residuals.

Given the low concentrations in the fine earth, the PLSR analysis was performed on the silt + clay fraction, despite it represents a small part (0.26–12%) of the bulk soil. A summary of the values for the elements in the predictors’ matrix can be found in Table 2. A PLSR model with three latent variables (LV) accounted for 72% of Hg variance (Fig. 1 SM), corresponding 41% to LV1, 14% to LV2 and 17% to LV3 (Table 3). The model regression coefficients were 0.32 β1, 0.31 β2, 0.73 β3 (Table 3). Figure 1 shows the relative weight of the LVs on the prediction of each sample. In general terms LV1 > LV2 > LV3, being the weight of LV3 relatively low for most of the samples. LV1 is characterised by positive loadings of Cu, Zn, and N and negative loading of Ca, Sr, and module (Table 4). LV2 shows positive loadings for P, C, Ca, and S and negative loadings for Ti and Fe (Table 4). LV3 is characterised by positive loadings for U, and S and negative loadings for P, and Mn (Table 4). Positive LV1 scores, were found for samples collected inside the burials, while negative scores were found for samples collected outside the burials (Fig. 2). LV2 positive scores mainly correspond to samples from burial T5, except for the most distal samples of the longitudinal transect of L07 (Fig. 2). LV3 scores show a more irregular pattern, showing positive scores on samples located in the thoracic area of the buried individuals (Fig. 2). The model residuals are low, with somewhat higher overestimation in the proximal samples of the L06 individual of T5 (Fig. 3).

**Table 2:** Summary of the elements selected as predictors (mg kg^−1^).

	Minimum	Median	Maximum	IQR
C	16,420	32,100	47,800	3215
N	662	1650	3335	273
P	0	2262	5150	1166
S	907	1289	2297	404
Ca	29,700	49,550	129,600	16,700
Ti	1900	3500	4100	575
Mn	613	1234	1482	141
Fe	31,300	39,750	46,800	3950
Cu	35	79	103	19
Zn	97	103	276	41
Sr	248	322	841	62
U	0	6	15	4

**Table 3:** PLSR summary. R^2^ = 0.72.

	LV1	LV2	LV3
Regression coefficients	0.32	0.31	0.73
% Variance explained	41%	14%	17%

**Table 4:** Matrix P (X-loadings).

	LV1	LV2	LV3
Cu	0.44	–	0.16
Zn	0.40	–	0.14
N	0.37	–	0.12
P	0.15	0.56	− 0.59
Mn	0.15	–	− 0.43
C	− 0.22	0.41	− 0.20
Ca	− 0.36	0.38	–
Sr	− 0.35	0.19	− 0.14
module	− 0.32	− 0.11	− 0.16
U	− 0.26	− 0.17	0.50
S	− 0.25	0.30	0.46
Ti	0.22	− 0.60	0.24
Fe	–	− 0.32	–

## Discussion

The low mercury concentrations of the fine earth samples are in agreement with previous studies in non-polluted soils^43–46^ and are consistent with the predominantly sandy grain size of the samples and the absence of mercury mineral phases. The silt + clay fraction, on the other hand, is usually enriched in organic matter and metals^47^.

The PLSR model obtained for the silt + clay fraction provided three main latent vectors, i.e. underlying processes, that account for a significant proportion (> 72%) of the variation in mercury concentrations in the two burials. LV1, which explains the largest amount of mercury variability (Table 3 and Fig. 1), accounts for differences between inside and outside the burials (Fig. 2). LV2 can be related to differences between the two burials (Fig. 2). LV3 seems to be due to within burial variability related to the proximity of the soil samples to areas where larger body mass (i.e. thoracic) was present (Fig. 2). Residuals showed no-clear pattern (Fig. 3) and can be linked to micro-scale soil variability.

**Figure 4:**
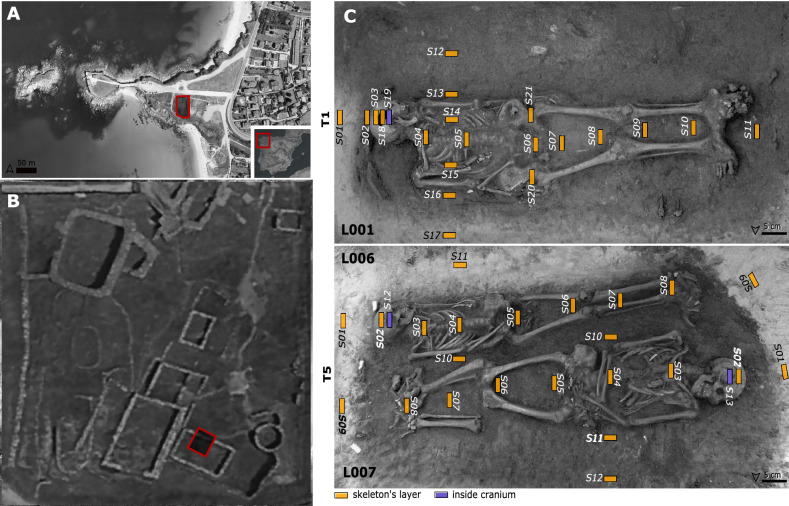
(**A**) and (**B**) Aerial view of A Lanzada with the approximated situation of the graves (**A**) modified from^79^, https://bit.ly/3FwpZrE; (B) modified from^80^, https://bit.ly/3BBqxKy. (**C**) Sampling model. In T1 the gray arrow represents the organic matter flow from inside the tomb to the surrounding area.

**Figure 5:**
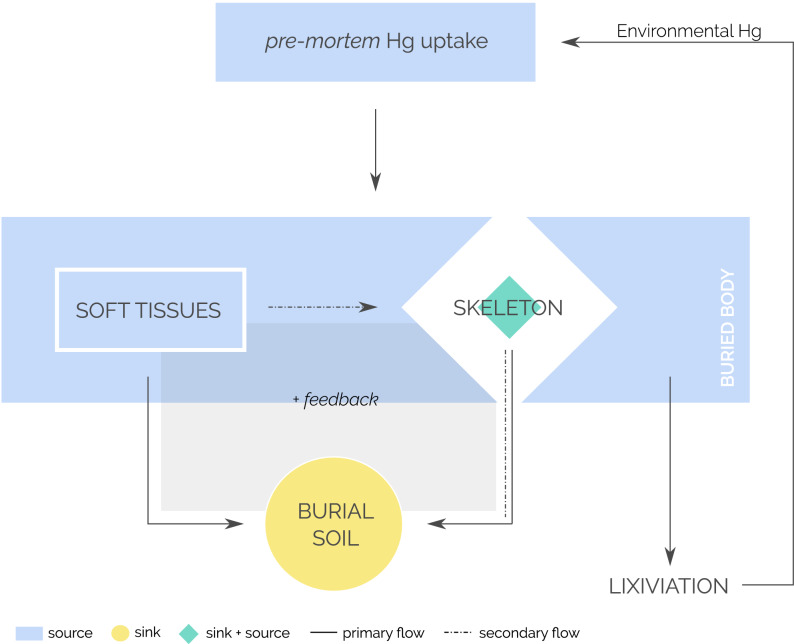
Mercury sink and sources from human exposure to burial/lixiviation in the soil through human body inhumation.

The LV1 variables with positive loadings (Cu, Zn, N) are related to the abundance of organic matter in the silt + clay fraction. Copper and Zn are known to bind to the organic matter (OM) and clay^48^ and N can be related to the total amount of OM^49,50^. Indicators with negative loadings include elements related to carbonates content (Ca and Sr) that can be linked to the presence of mollusc shells remains—note that the area is by the sea—and location within the burial (module). The positive regression coefficient for this LV indicates that mercury content increases with organic matter content, which is clearly higher inside the burials (Fig. 4C). Many investigations have shown that mercury in soils and sediments is correlated to organic matter content^14,51^. This can be seen as a sort of fingerprint of the individuals that also affected the mercury content (Fig. 5). The buried bodies most probably were both the source of the organic matter (together with the wood coffin) and the source of the mercury, which has accumulated in the finest and most reactive soil fractions (i.e. OM and clay minerals). The original substrate is a dune dominated by silicic sand with biogenic carbonates that lacks the capacity to retain mercury. This is consistent with the low Hg concentrations in the samples outside the burial environment (both in the fine earth: median = 1.6, IQR = 0.8 ng g^−1^; and the silt + clay fraction: median = 12.1, IQR = 3.6 ng g^−1^).

The LV2 indicators with positive loadings are P, C, and Ca, all constituents of the bone mineral component (i.e. hydroxyapatite), and S, which is a proxy of body decomposition. Among the indicators with negative loadings, Ti is indicative of soil fine fraction (i.e. clay)^52^. Iron is representative of major soil components (iron oxides) but also increases in the clay fraction as secondary oxy-hydroxides. LV2 scores are systematically positive in the double burial, T5, with the exception already mentioned before (distal samples in the L07 transect). This means that the fine soil fraction has higher concentrations of elements typical of the bone mineral matrix and S containing compounds than the same fraction in T1 burial. This difference most probably reflects the fact that T5 contains two individuals (an adolescent and a mature adult)—with a priori higher bone and soft tissue mass interacting with the soil components—and T1 contains only one individual (a single burial). The weight of LV2 in the samples is systematically positive in L06 individual, while it is negative in L01 and shows an irregular pattern in L07 (Fig. 1). The regression coefficient for LV2 is positive meaning that mercury content is higher in T5 burial compared to T1. The link between mercury and the other elements can be chemical bone alteration and sulphur compounds availability during early body decomposition.

Sulphur-containing amino acids (i.e. cysteine, cystine, and methionine) are subjected to desulphydralation by gastrointestinal microorganisms (i.e. sulphate-reducing bacteria) during body decomposition, producing the release of hydrogen sulphide gas, sulphides, ammonia, thiols, and pyruvic acids^49^. Given that during the main phase of tissues breakdown the tissue mass keeps anoxic^49^ and Hg tends to precipitate as its sulphide form^51^, HgS compounds can be expected in these environments. ^37^also hypothesised about this option, but provided no explanation for it. HgS compounds have extremely low solubility^51^, which will prevent from lixiviation, contributing to Hg retention in the burial environment. Thus, soft tissues decomposition may play a role in Hg release, as primary source (Fig. 5), but they are also key in providing compounds involved in Hg retention and accumulation in the burial environment.

Bone tissue acts as a sink for some elements during body decomposition^21^ and then as a source to the burial environment, altering the elements that are released and accumulated in the finer, more reactive, soil fractions. Indeed, bone is a potential primary and/or secondary source of mercury to the soil of the burial; while soft tissues are the primary source both to soil and bone (Fig. 5). A fact that is in agreement with the recent finding that mercury can be stabilised in bioapatite as (Hg)_3_(PO_4_)_2_^27^. Furthermore, it could explain why mercury concentration in archaeological bones is higher than the concentration found in actual bones from autopsies and biopsies^53,54^—although lower contents in recent bone can also be due to lower exposure as today environmental loads are much more controlled. The higher the bone mass the higher the transfer of mercury to the soil. ^42^also suggest that mercury distribution in the soil could be affected by burial context when graves are placed closed by as interactions between them cannot be excluded. In our case, T1 and T5 burials were far apart (~ 4 to 5 m) and with no other graves over or close to them. Another factor that should also be considered is that the overall mercury content of the individual in T1 compared to that of the individuals of T5 may have been different depending on environmental exposure during life. In a previous investigation, bone mercury content for individuals of the same period as those studied here varied between 4.6 and 299.9 ng g^−1^^13^.

The LV3 indicators with positive loadings are U and S. Uranium can be related to the geology of the area and has a high affinity for bone phosphates^55,56^, while S is a proxy for early stages of body decomposition, as previously mentioned in LV2 interpretation. Indicators with negative loadings are P, an indicator of bone alteration^55^, and Mn, a proxy for redox conditions^57^. The majority of samples with positive LV3 scores corresponds to those collected in the thoracic area of the three skeletons. The weight of LV3 tends to positive in samples of burial T5 while it does not show the same pattern in burial T1 (Fig. 1). This can be reflecting difference between burials. As already commented, the most important mercury target organs (kidneys, liver) are located close to the thoracic area^5^, which also contains the higher body mass—in comparison for example with the limbs or the head. Thus, differences in mercury content between soil close to thoracic area and soil close to the rest of the body would be expected. Furthermore, proteins forming the kidneys and liver start its putrefactive changes at early stages of body decomposition, while epidermis and muscles are more resistant to breakdown^49^. It is also likely that the higher content of Hg in this area is related with the higher availability during early body decomposition of both mercury and S compounds. Since the process takes place in the earliest stages of body decomposition the non-putrefied tissues would produce a confined environment and decrease mercury leaching. The negative loadings of P in this LV may also support this interpretation, as the soft tissue/bone ratio should also be higher in this body area and thus with lower net transfer of P to the soil. We speculate that “thoracic area” effect may be even more pronounced in graves with high mercury levels (i.e. periods with higher mercury levels of pollution and/or individuals which suffered an acute intoxication, among other causes).

As already commented, the model residuals showed no clear pattern (Fig. 3) and can reflect soil microscale heterogeneity.

Diagenesis is of major concern when trace elements are studied in archaeological bones, as there is the potential for the soil to be a source of elements for bones^30,55^. But works which assessed mercury diagenetic incorporation into bone in archaeological burial environment^22,23,31,41,42^ conclude that mercury bone levels were no related with diagenesis, unless the soils were naturally enriched in mercury. In a previous investigation in the same archaeological site we already reported very low mercury contents in soil (a pedo-sedimentary sequence produce an average of 0.9 ± 0.7 ng g^−1^) in comparison to bones of individuals of the same period as those of the present study (20.6 ± 23.5 ng g^−1^)^13^. Our PLSR model also supports the idea that the soil was not a significant source of Hg for buried bones. The situation seems to be rather the opposite; the buried individuals were most probably the source of the mercury accumulated in the soil (Fig. 5). In the particular case of our study, the decomposition of the buried bodies seems to have triggered a number of processes—developing a positive feedback mechanism—that lead to the rapid evolution of the parent soil material (i.e. sand dune), contributing to mineral weathering (i.e. decarbonation), clay formation and organic matter enrichment, creating the necessary conditions for the retention of the mercury released during the decomposition of the target organs (i.e. liver and kidney).

## Conclusions

In the present research we approached mercury distribution in burial environment through PLSR modelling of elemental composition of soil/sediment samples. The fitted model explains 72% of mercury variability. We have obtained three main conclusions. i) Our data confirm human bodies (soft tissues and bones) as sources of mercury in burial environments, as well as sources of organic matter which also contributes to mercury fixation. Soil seems to have not been a source of mercury for the skeletons, since mercury levels in samples surrounding the burials were very low when compared with those inside burials. In addition, ii) burial context seems to affect mercury distribution. We found differences that could be explained by the number of individuals buried together, as the body mass (i.e.: soft tissues and bones) is related to mercury content in the burial soil. iii) The thoracic area seems to produce a further local enrichment in Hg, most probably due to its release from target organs and its retention in the reactive soil fractions (organic matter and clay).

The obtained findings remark the importance of analysing soil/sediment associated to the burials in order to provide complementary information that can be key to understand some processes. Regarding the analytical procedure, we much encourage to analyse the finer (silt + clay) fractions when researching sandy soil/sediments, because this fraction is the most reactive and has better potential to contain information related to the body/soil interactions. In addition, the study of individuals/sites with low mercury levels can produce high quality results and complement those made in high polluted environment.

According to our results, soil/sediments from cemeteries can be significantly enriched in mercury compare with nearby ones. A fact that should be taken into account when describing soil properties or when those areas are having a new agrarian use. In addition, to assess the mercury content of an archaeological soil from a necropolis the proximity to the thoracic area of the skeletons can bias the obtained results.

Further research in mercury on soil/sediments associated to skeletons could help to achieve a better understanding of how mercury is distributed in these particular environments. Other questions such as how different burial contexts can affect mercury distribution in graves and how soft tissues affect it, remain open. Sulphur compounds, possibly related to body´s amino acids like methionine and cysteine, seem to contribute to Hg fixation—to analytically find these HgS compounds would be a necessary step in future research. HgS compounds are highly stable, limiting mercury release to the environment– even when the total amount is small. Therefore, to identify these compounds can help to prevent mercury release in individuals/cemeteries with high mercury concentrations.

## Material and methods

### Soil/sediment sampling

Soil and sediment samples of two post-Roman (AD 5th century) burials (T1 and T5) from A Lanzada site (Sanxenxo, Pontevedra, NW Spain, 42° 25′ 46″N 8° 52′ 25″W) (Fig. 4) have been analysed. A Lanzada is a well-known archaeological site with a widely studied necropolis (see among others^13,20,55,58–63^). However, there is only an unpublished work about soil/sediment geochemistry^64^. Geologically, A Lanzada site is a dune that was subject to anthropic alteration—at least since the BC 2nd century—that reduced its dimensions^65^. The site was discovered in the 18th century and was mainly excavated during the 1960s and 1970s. Most of the effort during the earlier excavations was done in the necropolis. It is placed in the East side of the site and has two funerary areas—Roman and post-Roman^62^. Neither a pedological study nor sediment-soil sampling was developed during those campaigns and information about the soil characteristics where bodies were inhumated was based on photographs and colour changes in the bones (see^55^). Skeletons were found in both dune sands and *earth* (i.e. acidic soils) independently of the funerary area^55^. Little effort was performed in the settlement area placed on the West side of the site, but recent campaigns in 2010 and 2016–2017 were mainly developed here.

The last excavation campaign took place in 2016–2017 and comprised the settlement area with houses occupied from the Late Bronze Age (~18 century BC) to the Late Iron Age-Early Roman Period (~ 1st century BC-AD). On the East margin of the excavation area a more monumental structure was discovered and dated to post-Roman times according to the archaeological material (~ 6th to 7th century AD). It was preliminary described as a church due to the monumental size of the walls^65^. Two coffin burials were discovered close to this structure and belonging to the same archaeological layer—named as T1 and T5. They were West–East oriented and radiocarbon dating indicates the individuals died between the 5th to the 6th centuries cal. AD. Both burials seem not to be related to the funerary areas previously found. T1 is a single burial containing a skeleton from an elder (> 60 years) female (L01). T5 is a multiple coetaneous burial containing two male skeletons, an adolescence (15–19 years) placed West–East (L06) and a mature adult (40–50 years) placed East–West (L07). All were in supine position with stretched arms and legs. Bodies were deposited presumably inside wood boxes (i.e. coffins) since nails were found surrounding the skeletons. The three skeletons were well-preserved, presenting the following abrasion degrees (according to^66^): L01 degree 3, L06 degree 2, L07 degree 2. Despite L06 and L07 low superficial abrasion degree and general good preservation, these skeletons have intense physical (pressure) and chemical alteration in localised areas (L06: face and feet, L07: long bones epiphyses), which results in the loss of some of the bones/bone areas^67^.

Both burials were excavated in the dune. A clear colour pattern of rectangular shape was evident in both burials, marking the area delimited by the wood coffins (the nails were found at the edges). The colour is most probably related with increased organic matter content (Fig. 4C). The sampling design consisted of one longitudinal and one transverse transect on each individual plus some additional samples taken from the inside the crania, and on L001 coxal area (Fig. 4). All samples have been taken on the field except for the additional ones, which were collected from soil adhered to the bones once in the laboratory. In total 46 soil/sediment samples were collected and analysed. Given that sample position is susceptible to affect mercury concentration, it was recorded using the longitudinal axis of each skeleton (Fig. 4). Sample coordinates were refereed to this reference axis and module was used as predictive variable.

### Elemental composition analysis

Mercury content was analysed in two fractions, fine earth (< 2 mm, FE) and silt + clay (< 0.05 mm, SC). The samples were previously dried (25 °C) and finely milled (< 0.5 mm). Mercury concentrations were determined using a DMA-80 (Mileston) hosted at the laboratory of the *Ecotoxicoloxía e Ecofisioloxía Vexetal* research group (*Departamento de Bioloxía Funcional,* USC) following the protocol described in^13^. BCR277R (128 ± 17 ng g^−1^) was used as certified reference material. The quantification limit was 0.51 ng g^−1^. Mean recovery for BCR27R was 91% (116 ± 17 ng g^−1^). Quality control has been done in 13 samples, reporting a relative error average of 6.37% for silt + clay.

Elemental composition analysis was carried out on dried (25 °C) silt + clay samples using two energy dispersive X-ray fluorescence (EMMA-XRF) analysers^68,69^ hosted at the RIAIDT facility of the *Universidade de Santiago de Compostela* (USC). This non-destructive technique provided concentration measurements for 10 elements (P, S, Ca, Ti, Mn, Fe, Cu, Zn, Sr, and U; see Table SM 1 for detection limits). Carbon and N analyses were done using a LECO (TruSpec CHNS) hosted at the Elemental Analysis Service of the RIAIDT in *Campus Terra* (USC).

Nitrogen was selected as a direct proxy for organic matter content (OM); Cu and Zn as indicators of metal enrichment in soil fine fractions bounded to OM and clay; Ca and Sr as proxies of biogenic carbonates, due to the presence of mollusc shells. The association of Ca, P and C is an indicator of the presence of bone, as these elements are the major constituents of bone mineral component (i.e. hydroxyapatite). Sulphur is used as proxy for OM forming strong bindings with Hg; Fe a major soil component; Ti is a proxy for fine grain fractions, and Mn is used as a proxy for red-ox conditions. Finally, U is characteristic of the geology of the area and presents high affinity for phosphate groups (i.e. bone mineral matrix).

### Statistical methods: partial least squares regression modelling

To understand mercury distribution in the soil we used Partial Least Squares Regression (PLSR)^70^. The elemental composition data set (elemental concentrations in the silt + clay fraction; see explanation in the results section) was used as predictors while Hg concentration was set as response variable. Zero imputation has been performed for those cases where the element concentration was below the detection limit following the robust method proposed by^71^. Due to the compositional nature of our data the data set was transformed to centered log ratios (clr)^72^ and scaled^70^. The main purpose of the PLSR modelling was the identification of the latent variables (i.e. processes) that affect Hg distribution and their weight in the soil samples. The model was performed using leave one out cross-validation without splitting the data in training and test sets to avoid the loss of relevant information and its explanatory power.

Statistical analyses and graphs were made using software R^73^, packages: {robCompositions} for zero imputation using ‘lmrob’ method^74^, {rgr} for clr transformation^75^, {plsRglm} for PLSR model^76^, {ggplot2} + {ggpubr} for graphics^77,78^.

See SM for: PLSR data set, ŷ values, T matrix and W* matrix.

## Supplementary Information

Supplementary Information.

## Data Availability

The datasets generated during and/or analysed during the current study are available from the corresponding author on reasonable request.

## References

[1] Evers D, Dellasala DA, Goldstein MI (2018). The effects of methylmercury on wildlife: A comprehensive review and approach for interpretation. Encyclopedia of the Anthropocene.

[2] Morel FMM, Kraepiel AML, Amyot M (1998). The chemical cycle and bioaccumulation of mercury. Ann. Rev. Ecol. Syst..

[3] Pushie MJ, Pickering IJ, Korbas M, Hackett MJ, George GN (2014). Elemental and chemically specific X-ray fluorescence imaging of biological systems. Chem. Rev..

[4] WHO. Exposure to Mercury: a Major Public Health Concern. (2007).

[5] Berlin M, Zalups RK, Fowler BA, Nordberg GF, Fowler BA, Nordberg M (2015). Chapter 46—Mercury. Handbook on the Toxicology of Metals (Fourth Edition).

[6] Clarkson TW (1997). The Toxicology of mercury. Crit. Rev. Clin. Lab. Sci..

[7] Abass K (2018). Quantitative estimation of mercury intake by toxicokinetic modelling based on total mercury levels in humans. Environ. Int..

[8] Liu G, Cai Y, O’Driscoll N, Feng X, Jiang G, Liu G, Cai Y, Driscoll N (2011). Overview of mercury in the environment. Environmental Chemistry and Toxicology of Mercury.

[9] García F, Ortega A, Domingo JL, Corbella J (2001). Accumulation of metals in autopsy tissues of subjects living in Tarragona county, Spain. J. Environ. Sci. Health Part A.

[10] Clarkson TW, Magos L (2006). The toxicology of mercury and its chemical compounds. Crit. Rev. Toxicol..

[11] Holmes P, James KAF, Levy LS (2009). Is low-level environmental mercury exposure of concern to human health?. Sci. Total Environ..

[12] Pasetto R, Martin-Olmedo P, Martuzzi M, Iavarone I (2016). Exploring available options in characterising the health impact of industrially contaminated sites. Ann. Ist Super Sanita.

[13] Álvarez-Fernández N, Martínez Cortizas A, López-Costas O (2020). Atmospheric mercury pollution deciphered through archaeological bones. J. Archaeol. Sci..

[14] Cooke CA, Martínez-Cortizas A, Bindler R, Sexauer Gustin M (2020). Environmental archives of atmospheric Hg deposition—A review. Sci. Total Environ..

[15] Leblanc M, Morales JA, Borrego J, Elbaz-Poulichet F (2000). 4,500-year-old mining pollution in southwestern Spain: Long-term implications for modern mining pollution. Econ. Geol..

[16] Cooke CA, Balcom PH, Biester H, Wolfe AP (2009). Over three millennia of mercury pollution in the Peruvian Andes. PNAS.

[17] Hunt Ortiz, M. A., Consuegra, S., Díaz del Río, P., Hurtado Pérez, V. & Montero Ruiz, I. *Neolithic and Chalcolithic –VI to III millennia BC– use of cinnabar (HgS) in the Iberian Peninsula: analytical identification and lead isotope data for an early mineral exploitation of the Almadén (Ciudad Real, Spain) mining district*. (2011).

[18] Martínez-Cortizas, A., Pontevedra-Pombal, X., García-Rodeja, E., Nóvoa-Muñoz, J. C. & Shotyk, W. Mercury in a Spanish peat bog: Archive of climate change and atmospheric metal deposition. *Science* **284**, 939–942 (1999). 10.1126/science.284.5416.939 10320369

[19] Martínez Cortizas, A., Peiteado Varela, E., Bindler, R., Biester, H. & Cheburkin, A. Reconstructing historical Pb and Hg pollution in NW Spain using multiple cores from the Chao de Lamoso bog (Xistral Mountains). *Geochimica et Cosmochimica Acta* **82**, 68–78 (2012).

[20] López-Costas O (2020). Human bones tell the story of atmospheric mercury and lead exposure at the edge of Roman World. Sci. Total Environ..

[21] Hedges REM (2002). Bone diagenesis: an overview of processes. Archaeometry.

[22] Yamada M (1995). Accumulation of mercury in excavated bones of two natives in Japan. Sci. Total Environ..

[23] Emslie SD (2015). Chronic mercury exposure in Late Neolithic/Chalcolithic populations in Portugal from the cultural use of cinnabar. Sci. Rep..

[24] Alexandrovskaya E, Alexandrovskiy A (2005). Radiocarbon data and anthropochemistry of ancient Moscow. Geochronometria.

[25] Ávila A, Mansilla J, Bosch P, Pijoan C (2014). Cinnabar in mesoamerica: poisoning or mortuary ritual?. J. Archaeol. Sci..

[26] Bocca B (2018). Metals in bones of the middle-aged inhabitants of Sardinia island (Italy) to assess nutrition and environmental exposure. Environ. Sci. Pollut. Res..

[27] Cervini-Silva, J., Muñoz, M. de L., Palacios, E., Ufer, K. & Kaufhold, S. Natural incorporation of mercury in bone. *J. Trace Elements Med. Biol.* **67**, 126797 (2021). 10.1016/j.jtemb.2021.126797 34087580

[28] Cervini-Silva, J., Muñoz, M. de L., Palacios, E., Jimenez-Lopez, J. C. & Romano-Pacheco, A. Ageing and preservation of HgS-enriched ancient human remains deposited in confinement. *J. Archaeol. Sci.: Rep.* **18**, 562–567 (2018).

[29] Cervini-Silva J (2013). Cinnabar-preserved bone structures from primary osteogenesis and fungal signatures in ancient human remains. Geomicrobiol. J..

[30] Emslie SD (2019). Mercury in archaeological human bone: biogenic or diagenetic?. J. Archaeol. Sci..

[31] Kepa M (2012). Analysis of mercury levels in historical bone material from syphilitic subjects–pilot studies (short report). Anthropol. Anz..

[32] Ochoa-Lugo M (2017). The effect of depositional conditions on mineral transformation, chemical composition, and preservation of organic material in archaeological Hg-enriched bone remains. J. Archaeol. Sci.: Rep..

[33] Panova TD, Dmitriev AYu, Borzakov SB, Hramco C (2018). Analysis of arsenic and mercury content in human remains of the 16th and 17th centuries from Moscow Kremlin necropolises by neutron activation analysis at the IREN facility and the IBR-2 reactor FLNP JINR. Phys. Part. Nuclei Lett..

[34] Rasmussen KL (2021). Investigations of the relics and altar materials relating to the apostles St James and St Philip at the Basilica dei Santi XII Apostoli in Rome. Herit. Sci..

[35] Rasmussen KL (2020). Comparison of trace element chemistry in human bones interred in two private chapels attached to Franciscan friaries in Italy and Denmark: An investigation of social stratification in two medieval and post-medieval societies. Heritage Sci..

[36] Rasmussen KL (2017). On the distribution of trace element concentrations in multiple bone elements in 10 Danish medieval and post-medieval individuals. Am. J. Phys. Anthropol..

[37] Rasmussen KL, Skytte L, Jensen AJ, Boldsen JL (2015). Comparison of mercury and lead levels in the bones of rural and urban populations in Southern Denmark and Northern Germany during the Middle Ages. J. Archaeol. Sci.: Rep..

[38] Rasmussen KL (2013). Was he murdered or was he not?—Part I: Analyses of mercury in the remains of Tycho Brahe. Archaeometry.

[39] Rasmussen KL (2013). The distribution of mercury and other trace elements in the bones of two human individuals from medieval Denmark—The chemical life history hypothesis. Herit. Sci..

[40] Torino M (2015). Convento di San Francesco a Folloni: The function of a Medieval Franciscan Friary seen through the burials. Herit. Sci..

[41] Walser JW, Kristjánsdóttir S, Gowland R, Desnica N (2019). Volcanoes, medicine, and monasticism: Investigating mercury exposure in medieval Iceland. Int. J. Osteoarchaeol..

[42] Rasmussen KL (2008). Mercury levels in Danish Medieval human bones. J. Archaeol. Sci..

[43] Armesto AG (2018). Total mercury distribution among soil aggregate size fractions in a temperate forest podzol. Span. J. Soil Sci..

[44] do Valle, C. M., Santana, G. P., Augusti, R., Egreja Filho, F. B. & Windmöller, C. C. Speciation and quantification of mercury in Oxisol, Ultisol, and Spodosol from Amazon (Manaus, Brazil). *Chemosphere* **58**, 779–792 (2005). 10.1016/j.chemosphere.2004.09.005 15621191

[45] Fiorentino JC, Enzweiler J, Angélica RS (2011). Geochemistry of mercury along a soil profile compared to other elements and to the parental rock: Evidence of external input. Water Air Soil Pollut..

[46] Roulet, M. *et al.* The geochemistry of mercury in central Amazonian soils developed on the Alter-do-Chão formation of the lower Tapajós River Valley, Pará state, Brazil1The present investigation is part of an ongoing study, the CARUSO project (IDRC-UFPa-UQAM), initiated to determine the sources, fate, and health effects of MeHg in the Lower Tapajós area.1. *Sci. Total Environ.* **223**, 1–24 (1998). 10.1016/s0048-9697(98)00265-4 9850600

[47] Qin F (2014). Evaluation of trace elements and identification of pollution sources in particle size fractions of soil from iron ore areas along the Chao River. J. Geochem. Expl..

[48] Acosta JA, Martínez-Martínez S, Faz A, Arocena J (2011). Accumulations of major and trace elements in particle size fractions of soils on eight different parent materials. Geoderma.

[49] Janaway RC, Percival SL, Wilson AS, Percival SL (2009). Decomposition of Human Remains. Microbiology and Aging: Clinical Manifestations.

[50] Obrist D, Johnson DW, Lindberg SE (2009). Mercury concentrations and pools in four Sierra Nevada forest sites, and relationships to organic carbon and nitrogen. Biogeosciences.

[51] Schuster E (1991). The behavior of mercury in the soil with special emphasis on complexation and adsorption processes—A review of the literature. Water Air Soil Pollut..

[52] Taboada T, Cortizas AM, García C, García-Rodeja E (2006). Particle-size fractionation of titanium and zirconium during weathering and pedogenesis of granitic rocks in NW Spain. Geoderma.

[53] Babuśka-Roczniak M (2021). Occurrence of mercury in the knee joint tissues. Pol. Ann. Med..

[54] Domingo JL, García F, Nadal M, Schuhmacher M (2017). Autopsy tissues as biological monitors of human exposure to environmental pollutants. A case study: Concentrations of metals and PCDD/Fs in subjects living near a hazardous waste incinerator. Environ. Res..

[55] López-Costas, O., Lantes-Suárez, Ó. & Martínez Cortizas, A. Chemical compositional changes in archaeological human bones due to diagenesis: Type of bone vs soil environment. *J. Archaeol. Sci.* **67**, 43–51 (2016).

[56] Taboada, T., Martínez Cortizas, A., García, C. & García-Rodeja, E. Uranium and thorium in weathering and pedogenetic profiles developed on granitic rocks from NW Spain. *Sci. Total Environ.* **356**, 192–206 (2006). 10.1016/j.scitotenv.2005.03.030 15923024

[57] Windmöller, C. C., Durão, W. A., de Oliveira, A. & do Valle, C. M. The redox processes in Hg-contaminated soils from Descoberto (Minas Gerais, Brazil): Implications for the mercury cycle. *Ecotoxicol. Environ. Saf.* **112**, 201–211 (2015). 10.1016/j.ecoenv.2014.11.009 25463872

[58] Blanco Freijeiro, A., Fusté Ara, M. & García Alén, A. La necrópolis galaico-romana de La Lanzada (Noalla, Pontevedra) II. *Cuadernos de estudios gallegos* **22**, 5–23 (1967).

[59] Blanco Freijeiro, A., Fusté Ara, M. & García Alén, A. La necrópolis galaico-romana de La Lanzada (Noalla, Pontevedra). *Cuadernos de estudios gallegos* **16**, 141–158 (1961).

[60] Kaal, J., López-Costas, O. & Martínez Cortizas, A. Diagenetic effects on pyrolysis fingerprints of extracted collagen in archaeological human bones from NW Spain, as determined by pyrolysis-GC-MS. *J. Archaeol. Sci.* **65**, 1–10 (2016).

[61] López Costas, O. Antropología de los restos óseos humanos de Galicia: estudio de la población romana y medieval gallega. (Universidad de Granada, 2012).

[62] López-Costas, O. Taphonomy and burial context of the Roman/post-Roman funerary areas (2nd to 6th centuries AD) of A Lanzada, NW Spain. *Estudos do Quaternário/Quaternary Studies* 55–67 (2015) 10.30893/eq.v0i12.111.

[63] López-Costas O, Müldner G (2016). Fringes of the empire: Diet and cultural change at the Roman to post-Roman transition in NW Iberia. Am. J. Phys. Anthropol..

[64] García López, Z., López Costas, O. & Martínez Cortizas, A. Análisis de sedimentos asociados a restos humanos de la Necrópolis de A Lanzada y Adro Vello (Pontevedra). (2019).

[65] Rodríguez Martínez, R. M. Informe valorativo da intervención arqueolóxica para a recuperación patrimonial do xacemento de A Lanzada (Sanxenxo, Pontevedra). Fase II. (2017).

[66] Brickley, M. & McKinley, J. I. Determination of sex from archaeological skeletal material and assessment of parturition. in *Guidelines to the Standards for Recording Human Remains.* 23–25 (BABAO, Dept. of Archaeology, University of Southampton. Institute of Field Archaeologist, University of Reading, 2004).

[67] López Costas, O. *et al.* Informe final: Estudio de esqueletos humanos y de secuencias edafo-sedimentárias del yacimiento de A Lanzada. En: Rodríguez Martínez, R.M., 2017. Informe valorativo da intervención arqueolóxica para a recuperación patrimonial do xacemento de A Lanzada (Sanxenxo, Pontevedra). Fase II. (2017).

[68] Cheburkin AK, Shotyk W (1999). Determination of trace elements in aqueous solutions using the EMMA miniprobe XRF analyzer. X-Ray Spectrom..

[69] Cheburkin AK, Shotyk W (1999). High-sensitivity XRF analyzer (OLIVIA) using a multi-crystal pyrographite assembly to reduce the continuous background. X-Ray Spectrom..

[70] Wold S, Sjöström M, Eriksson L (2001). PLS-regression: a basic tool of chemometrics. Chemometrics Intell. Lab. Syst..

[71] Martín-Fernández JA, Hron K, Templ M, Filzmoser P, Palarea-Albaladejo J (2012). Model-based replacement of rounded zeros in compositional data: Classical and robust approaches. Comput. Stat. Data Anal..

[72] Egozcue JJ, Pawlowsky-Glahn V, Mateu-Figueras G, Barceló-Vidal C (2003). Isometric logratio transformations for compositional data analysis. Mathe. Geol..

[73] R Core Team. *R: A Language and Environment for Statistical Computing*. (R Foundation for Statistical Computing, 2021).

[74] Filzmoser P, Hron K, Templ M (2018). Applied Compositional Data Analysis. With Worked Examples.

[75] Garrett, R. G. *rgr: Applied Geochemistry EDA*. (2018).

[76] Bertrand, F. & Maumy-Bertrand, M. *Partial Least Squares Regression for Generalized Linear Models*. (2019).

[77] Kassambara, A. *ggpubr: ‘ggplot2’ Based Publication Ready Plots*. (2020).

[78] Wickham H (2016). ggplot2: Elegant Graphics for Data Analysis.

[79] Punta A Lanzada, O Grove (Galicia, Spain) 42°25′44.61″N 8°52′29.31″W elev 16 m eye alt 585m. Google Earth. Jully 18, 2020. March 20, 2021. https://bit.ly/3FwpZrE.

[80] A Lanzada site (Galicia, Spain) 42°25′44.64″N 8°52″29.42″W elev 16m eye alt 549m. Google Earth. Jully 18, 2020. October 12, 2021. https://bit.ly/3BBqxKy.

