# Supplementary material for: Approaching mercury distribution in burial environment using PLS-R modelling
Source: Sci Rep. 2021 Oct 27;11:21231. doi: 10.1038/s41598-021-00768-8 (PMC8551184; doi:10.1038/s41598-021-00768-8)
Supplement: Supplementary file 1 — Supplementary Information. [file 41598_2021_768_MOESM1_ESM.pdf]

Approaching mercury distribution in burial environment using PLS-R modelling

Noemi Álvarez-Fernández[1,*], Antonio Martínez Cortizas[1], Zaira García-López[1], Olalla López-Costas[2,3,4].

[1]CRETUS, EcoPast (GI-1553), Universidade de Santiago de Compostela, 15782, Spain.
[2]EcoPast (GI-1553), CRETUS, Archaeology Department of History, Universidade de Santiago de Compostela, 15782, Spain.
[3]Archaeological Research Laboratory, Stockholm University, Wallenberglaboratoriet, SE-10691, Sweden.
[4]Laboratorio de Antropología Física, Facultad de Medicina, Universidad de Granada, 18012, Spain.

*n.alvarez.fernandez@usc.es

SUPPLEMENTARY MATERIAL

Table SM1. Detection limits (mg kg^{-1})

C	N	P	S	Ca	Ti	Mn	Fe	Cu	Zn	Sr	Hg	U
20	20	0.1	100	200	5	100	100	4	5	5	$1.53 \cdot 10^{-4}$	1

Table SM 2. PLSR data set.

Hg	N	S	P	Zn	Cu	Sr	Ca	C	Fe	U	Ti	Mn	module
-10.26	1.30	1.64	1.51	-0.76	-2.11	0.39	5.63	4.67	4.54	-3.93	1.77	1.08	95.52
-10.15	1.45	1.22	1.69	-0.70	-1.76	-0.28	4.69	4.32	4.66	-3.72	2.23	1.06	87.57
-10.02	1.41	1.09	1.33	-0.56	-1.61	-0.25	4.76	4.36	4.69	-4.96	2.29	1.16	75.66
-10.22	1.34	1.10	1.94	-0.61	-1.63	-0.31	4.68	4.27	4.61	-3.80	2.26	1.11	55.00
-9.88	1.49	0.94	1.67	-0.53	-1.56	-0.25	4.75	4.38	4.64	-3.81	2.17	1.11	20.00
-10.09	1.58	1.10	1.59	-0.58	-1.69	-0.27	4.68	4.41	4.61	-4.47	2.28	1.12	0.00
-9.88	1.51	1.06	1.72	-0.57	-1.54	-0.34	4.66	4.33	4.62	-4.22	2.29	1.12	10.00
-10.22	1.50	1.00	1.39	-0.65	-1.71	-0.34	4.62	4.34	4.56	-3.58	2.29	1.16	30.07
-9.98	2.09	1.12	1.53	-0.61	-1.57	-0.41	4.50	4.33	4.61	-3.71	2.25	1.11	45.10
-10.01	1.54	0.99	0.83	-0.53	-1.68	-0.25	4.80	4.42	4.67	-4.26	2.25	1.19	75.17
-10.14	1.32	1.05	1.94	-0.76	-1.71	0.12	5.19	4.46	4.57	-3.87	1.97	1.12	100.12
-10.08	1.28	1.19	2.53	-0.62	-1.75	0.10	5.12	4.50	4.68	-3.93	2.00	1.20	46.10
-9.38	1.98	1.62	1.08	-0.11	-1.14	0.22	5.34	4.87	5.11	-5.18	2.26	1.66	37.20
-9.84	1.40	1.37	0.99	-0.51	-1.45	-0.23	4.82	4.35	4.71	-3.94	2.15	1.25	31.05
-9.53	1.72	1.74	1.48	-0.22	-1.24	-0.02	5.00	4.63	4.92	-9.14	2.53	1.46	33.11
-9.78	1.44	1.38	1.59	-0.54	-1.56	-0.30	4.73	4.36	4.62	-4.97	2.28	1.14	39.70
-9.78	1.30	1.26	1.27	-0.58	-1.56	-0.19	4.92	4.35	4.56	-4.40	2.21	1.04	50.00
-10.03	1.28	0.94	1.76	-0.70	-1.63	-0.45	4.47	4.22	4.60	-3.54	2.16	1.12	75.66
-9.97	1.60	0.97	1.90	-0.65	-1.56	-0.40	4.60	4.36	4.60	-4.01	2.20	1.12	75.66
-9.83	1.47	1.11	1.90	-0.59	-1.61	-0.36	4.58	4.32	4.66	-4.11	2.17	1.20	10.00
-9.88	1.61	1.09	1.95	-0.40	-1.50	-0.39	4.51	4.37	4.74	-5.67	2.24	1.29	10.00
-10.10	1.05	1.72	0.25	-0.79	-1.95	0.69	5.78	4.83	4.63	-3.94	1.68	1.21	80.00
-9.75	1.41	1.61	2.34	-0.79	-1.88	-0.06	5.22	4.51	4.41	-4.21	2.04	0.94	63.00
-9.40	1.35	1.27	2.22	-0.56	-1.69	-0.15	4.92	4.37	4.48	-4.15	2.13	0.93	40.05
-9.28	1.44	0.96	1.83	-0.46	-1.60	-0.20	4.84	4.38	4.59	-4.22	2.12	1.12	25.02
-9.81	1.55	1.37	1.55	-0.44	-1.56	-0.02	5.09	4.48	4.69	-5.18	2.18	1.22	0.00
-10.16	1.50	1.16	1.78	-0.69	-1.81	-0.11	4.99	4.43	4.50	-4.10	2.19	1.05	30.59
-9.98	1.57	1.66	1.58	-0.67	-1.79	-0.11	5.20	4.53	4.55	-4.75	2.09	1.06	50.99
-9.96	1.32	1.57	2.06	-0.68	-1.75	-0.02	5.17	4.46	4.59	-4.32	1.92	1.03	76.49
-10.40	0.79	1.78	1.75	-0.78	-1.96	0.77	5.81	4.81	4.60	-3.28	1.59	1.18	111.80
-9.94	1.42	1.14	2.03	-0.61	-1.61	-0.21	4.77	4.35	4.54	-4.62	2.22	1.05	32.02
-10.25	1.22	1.65	1.31	-0.86	-2.04	0.54	5.66	4.78	4.57	-4.28	1.91	1.20	30.81
-10.21	1.42	1.48	2.59	-0.44	-1.52	0.05	5.21	4.53	4.80	-9.22	2.35	1.27	87.00
-10.31	0.97	1.34	0.16	-0.74	-1.97	0.49	5.49	4.58	4.67	-3.66	2.15	1.22	110.00
-9.94	1.36	1.34	1.79	-0.65	-1.64	-0.28	4.88	4.41	4.57	-3.95	2.15	1.08	90.00
-9.46	1.31	1.24	2.40	-0.50	-1.57	-0.32	4.78	4.29	4.47	-4.71	2.07	0.98	60.21
-9.79	1.38	1.10	2.44	-0.51	-1.61	-0.36	4.76	4.27	4.45	-4.25	2.13	1.02	35.13
-9.63	1.40	0.98	1.76	-0.42	-1.50	-0.23	4.79	4.39	4.61	-4.34	2.13	1.20	0.00
-9.71	1.47	1.18	1.95	-0.24	-1.27	-0.03	4.91	4.49	4.81	-9.28	2.33	1.40	30.07
-9.68	1.69	1.14	1.95	-0.21	-1.16	-0.07	4.85	4.54	4.88	-9.25	2.39	1.47	55.90
-10.08	1.25	1.40	1.62	-0.74	-1.73	-0.18	4.82	4.30	4.61	-4.59	2.26	1.07	75.66
-10.09	1.38	1.69	-8.52	-0.55	-1.56	0.40	5.18	4.59	5.23	-5.48	2.74	1.30	110.45
-9.69	1.53	1.26	1.74	-0.54	-1.51	-0.22	4.92	4.42	4.60	-4.53	2.09	1.14	41.34
-10.02	1.32	0.97	1.70	-0.41	-1.49	-0.25	4.67	4.27	4.71	-4.79	2.13	1.21	43.60
-10.43	1.32	1.46	-8.78	-0.60	-1.82	0.33	5.02	4.42	5.10	-3.14	2.94	1.43	53.15
-9.82	1.65	1.38	2.11	-0.74	-1.71	-0.38	4.83	4.43	4.50	-4.06	2.16	1.06	90.00

Table SM 3. ŷ values.

ŷ
-10.19
-10.15
-10.07
-9.99
-9.82
-9.90
-9.82
-10.08
-9.80
-10.06
-10.24
-10.10
-9.42
-9.72
-9.58
-9.77
-9.76
-10.18
-10.02
-9.94
-9.84
-10.19
-9.88
-9.67
-9.78
-9.67
-10.01
-9.81
-9.86
-10.20
-9.86
-10.29
-10.03
-10.49
-9.91
-9.62
-9.69
-9.70
-9.78
-9.79
-10.12
-10.12
-9.70
-9.85
-10.40
-9.97

Table SM 4. Matrix T (X-scores).

LV1	LV2	LV3
-3.81	1.38	-0.25
-0.71	-1.18	-0.31
0.29	-0.99	-0.41
0.26	-0.85	-0.05
1.14	-0.14	0.14
1.15	-0.29	-0.23
1.47	-0.39	0.10
0.49	-1.12	-0.53
1.86	-0.53	0.09
0.30	-0.96	-0.38
-1.81	-0.21	-0.74
-0.86	0.34	-0.63
2.81	1.91	0.59
0.95	0.14	0.61
3.14	1.12	-0.03
0.93	0.03	0.40
0.10	0.18	0.78
-0.02	-1.60	-0.58
0.69	-0.87	-0.37
1.22	-0.52	-0.38
2.61	-0.29	-0.54
-4.42	1.75	-0.17
-1.79	1.26	0.52
0.02	0.84	0.98
1.10	0.24	0.21
1.26	1.03	0.36
-0.42	0.04	-0.22
-0.77	1.16	0.49
-1.46	1.09	0.52
-5.23	2.11	-0.01
0.58	-0.01	0.13
-3.50	1.33	-0.88
0.75	0.47	-1.06
-4.00	-0.79	-0.80
-0.59	-0.04	0.38
0.65	0.79	0.99
0.98	0.44	0.65
1.76	0.42	0.24
3.06	0.61	-0.82
3.56	0.35	-0.98
-1.03	-0.82	-0.18
-2.23	-3.11	1.34
0.88	0.71	0.49
1.42	-0.39	-0.03
-2.38	-4.61	0.56
-0.43	-0.04	0.01

Table SM 5. Matrix W* (model weights).

	LV1	LV2	LV3
**N**	0.35	0.07	-0.03
**S**	-0.11	0.37	0.40
**P**	0.24	0.34	-0.42
**Zn**	0.48	0.39	0.49
**Cu**	0.49	0.32	0.44
**Sr**	-0.30	0.03	-0.08
**Ca**	-0.22	0.29	0.12
**C**	-0.10	0.30	0.01
**Fe**	-0.02	-0.32	-0.15
**U**	-0.24	-0.04	0.41
**Ti**	0.05	-0.47	-0.03
**Mn**	0.05	-0.27	-0.56
**module**	-0.35	-0.20	-0.04

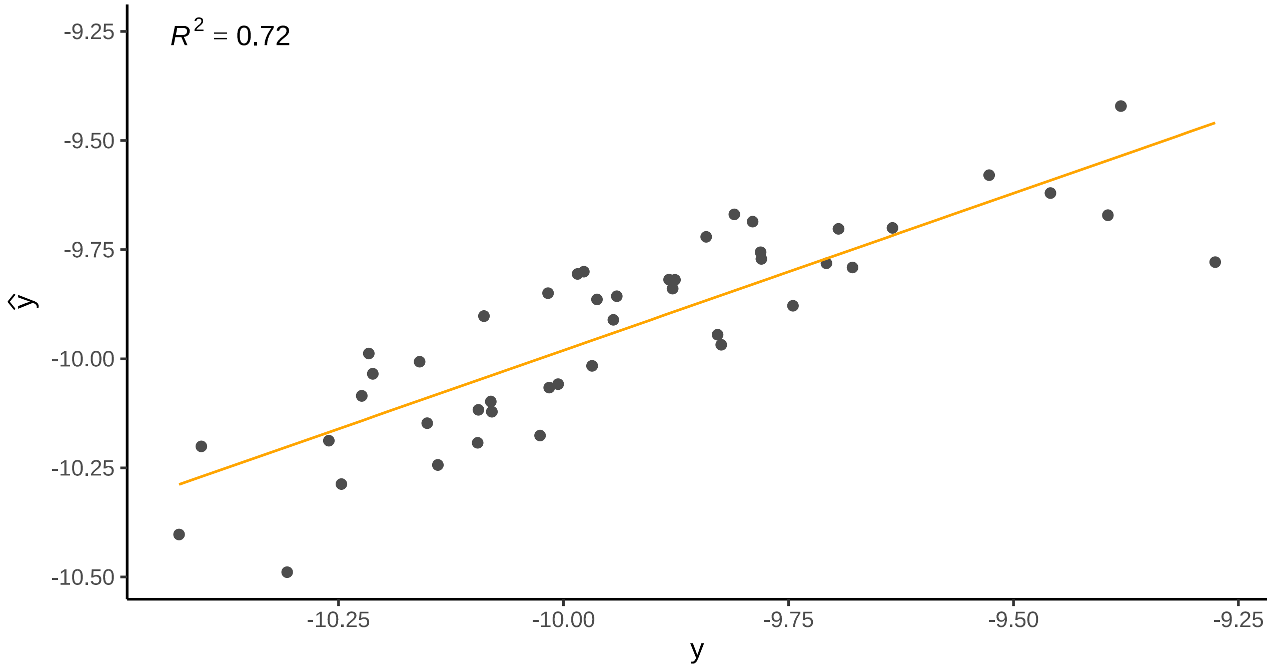

Figure 1 SM. PLSR response plot.
